# Effect of Lignin Modification on Properties of Kenaf Core Fiber Reinforced Poly(Butylene Succinate) Biocomposites

**DOI:** 10.3390/ma12244043

**Published:** 2019-12-05

**Authors:** Harmaen Ahmad Saffian, Kim Hyun-Joong, Paridah Md Tahir, Nor Azowa Ibrahim, Seng Hua Lee, Ching Hao Lee

**Affiliations:** 1 Institute of Tropical Forestry and Forest Products (INTROP), Universiti Putra Malaysia, UPM Serdang 43400, Selangor, Malaysia; leechinghao@upm.edu.my; 2 Laboratory of Adhesion & Bio-Composites, College of Agriculture and Life Sciences (CALS), Seoul National University, Seoul 08826, Korea; hjokim@snu.ac.kr; 3 Faculty of Science, Universiti Putra Malaysia, UPM Serdang 43400, Selangor, Malaysia; norazowa@upm.edu.my

**Keywords:** kenaf core, maleic anhydride, kraft lignin, extrusion, modification

## Abstract

In this study, the effects of lignin modification on the properties of kenaf core fiber reinforced poly(butylene succinate) biocomposites were examined. A weight percent gain (WPG) value of 30.21% was recorded after the lignin were modified with maleic anhydride. Lower mechanical properties were observed for lignin composites because of incompatible bonding between the hydrophobic matrix and the hydrophilic lignin. Modified lignin (ML) was found to have a better interfacial bonding, since maleic anhydrides remove most of the hydrophilic hydrogen bonding (this was proven by a Fourier-transform infrared (FTIR) spectrometer—a reduction of broadband near 3400 cm^−1^, corresponding to the –OH stretching vibration of hydroxyl groups for the ML samples). On the other hand, ML was found to have a slightly lower glass transition temperature, Tg, since reactions with maleic anhydride destroy most of the intra- and inter-molecular hydrogen bonds, resulting in a softer structure at elevated temperatures. The addition of kraft lignin was found to increase the thermal stability of the PBS polymer composites, while modified kraft lignin showed higher thermal stability than pure kraft lignin and possessed delayed onset thermal degradation temperature.

## 1. Introduction

Petroleum is a non-renewable resource unable to replenish itself at a rate suitable for sustainable economic extraction. Conservative estimates ascertain that this fossil fuel could be exhausted in 50 to 60 years, based on the current consumption rate [1]. As a result, petroleum-based plastics could face supply issues. Lignin, a renewable raw plastic material, thus, has high potential to replace conventional plastic in composites. Jayaramudu [2] reported that cellulose nanofiber (CNF) films are able to bind with poly(ethylene oxide)-lignin blends, exhibiting almost two-fold higher shear strength that those with pure poly(ethylene oxide). However, chemical modification is often needed to enhance the performance of pristine lignin. Allytrimethoxy silane (VPS)-modified lignin is used as a reinforcing filler for unsaturated polyester (UPE) resin. UPE composites reinforced with modified lignin reveal better tensile and impact properties owing to its enhanced interfacial adhesion [3]. The application of lignin can mitigate the strength loss of composites by weathering. Spiridon [4] fabricated polylactic acid (PLA)-based composites with the addition of lignin. On being subjected to accelerated weathering, the PLA/lignin composites showed only slight reduction in tensile and impact strength, compared to that of pure PLA.

Poly(butylene succinate) (PBS) polymer is fabricated by a polycondensation process involving succinic acid and 1,4-butanediol [5]. Attractive reasons to select PBS are its promising physical and mechanical properties with high biodegradability and degrees of processing [6,7]. PBS polymer is highly stable until about 350 °C, and starts degrading with mass loss at 390 °C. This proves that PBS has better thermal stability than the PLA polymer, which shows major mass loss at 365–385 °C [8].

Kenaf (*Hibiscus cannabinus*) is an annual fiber crop grown in Malaysia. The kenaf plant can grow to about 1.5 m in 120 days and its stem is made of bark and core fibers. The plant’s core resembles wood and makes up 60–70% of the stem’s dry weight; bark fibers contribute to the remaining 30–40%, and have a dense structure [9]. Several studies have reported the use of kenaf core fibers (KCF) as reinforcements in composite panels [10,11]. A study on KCF-reinforced unsaturated polyester composite revealed that 20 wt % of KCF reinforcements is the benchmark to enhance the composite’s mechanical properties [12]. Similar enhancement results were obtained by KCF/high density polyethylene (HDPE) composites [13,14]. The addition of KCF into the matrix reduces the chain mobility and consequently produces more rigid and tough composites. Besides, Joonobi [15] studied the thermal properties of KCF: the low thermal stability of KCF showed major thermal degradation at 300 °C, but left a high amount of residua after TGA testing, due to the presence of carbonaceous materials in the KCF (hemicellulose and lignin components).

Synthesized lignin can be used in various applications. In comparison to conventional synthetic materials, lignin have several advantages. For example, they are environment-friendly owing to their biodegradable and carbon neutral features. Besides, as an industrial by-product, lignin is readily available [16]. In addition, lignin has also been known to have antioxidant, antimicrobial, and stabilizer properties [17,18,19]. Therefore, the application of lignin as reinforcement for the fabrication of polymer composite could be beneficial. Nevertheless, related studies on this topic, particularly on the use of PBS as a matrix, is lacking. This study was thus conducted to examine the potential of lignin-based polymer composite production by the extrusion method. The objectives of this study were to investigate the effects of the incorporation of maleic anhydride-modified lignin on the mechanical and thermal properties of kenaf core fiber reinforced poly(butylene succinate) biocomposites.

## 2. Materials and Methods

### 2.1. Preparation of Materials

Kraft Lignin (low sulfonate) was purchased from Aldrich-Sigma, St. Louis, MO, USA. Poly(butylene succinate) (Bionolle 1020) (PBS) from Showa Highpolymers Co. Ltd., Tokyo, Japan; polymeric methylene diphenyl diisocyanate (PMDI) from Kumho Mitsui Chemicals, Seoul, Korea; and kenaf core fibre (KCF) from National Kenaf and Tabaco Council (LKTN), Kota Bharu, Malaysia, were used in this study.

### 2.2. Methods

#### 2.2.1. Chemical Modification of Kraft Lignin

The purchased kraft lignin was dried in an oven at 105 ± 3 °C overnight before modification by maleic anhydride (MA). Forty grams of MA were placed in a 250-mL round-bottom flask fitted in a conventional microwave oven. It was irradiated until a molten mass was produced. Twenty grams of pure kraft lignin was immersed in the molten MA in a separate setup—a heating mantle set at 100 °C for 3 min. Next, the mixture was put into a microwave oven for 20-min reflux heating with 30 s pulsed exposure. The products were then filtered and washed with an excess of distilled water. The washed products were then dried in a laboratory oven at 80 °C for 24 h.

Weight percent gain (WPG) was calculated after the modification was done to assess the extent of the reaction. Equation (1) was used to calculate WPG.

WPG (%) = (W_2_ − W_1_)/W_1_ × 100 (1)

where W_1_ is the oven dried weight of the sample (g) before modification and W_2_ is the weight of the samples (g) after modification.

#### 2.2.2. Composite Fabrication and Formulation

Before composite processing, ML, PBS, KCF, and PMDI were crushed to pellet size 3 mm in diameter and 5–6 mm long, and then dried at 103 ± 2 °C for 24 h using a convection oven. The pellets of the respective materials were then mixed according to formulations shown in Table 1. The mixtures of ML, PBS, KCF, and PMDI were processed in an internal mixer machine (BRABENDER, Duisburg, Germany) at 130 °C barrel temperature, 50 rpm screw rotation (co-rotation configuration), and compounded for 6 min. After compounding, the formulations were pressed in an extruder at 130 °C for 15 min.

#### 2.2.3. Characterization

The effect of the modified lignin (ML) on the PBS matrix was examined and characterised. A comparison was made between the properties of PBS composites fabricated with the modified and unmodified lignin. In the process, 10 wt % kenaf core fibre and 3 wt % PMDI as a compatibilizer were incorporated into the PBS composite. The prepared samples were then tested for mechanical and thermal properties. Five replicates and one replicate were used for testing of the mechanical and thermal properties, respectively. Details of the mentioned testing are described in the following sections.

##### Mechanical Testing

**Figure 1:**
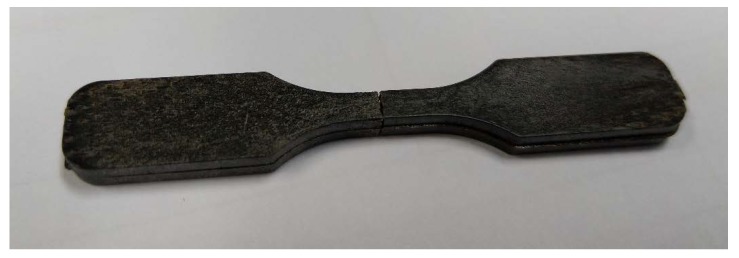
PBS/Lignin/PMDI/KCF composite after the tensile test.

The tensile properties of the composites produced were evaluated according to procedures specified in ASTM D 638 (American Society for Testing and Materials, West Conshohocken, PA, USA) and ASTM D 790 (American Society for Testing and Materials, West Conshohocken, PA, USA), respectively, under room temperature. The samples were tested using a Universal Testing Machine (Instron 3382, INSTRON, University Ave Norwood, MA, USA) equipped with Blue Hill software (Bluehill 2, INSTRON, University Ave Norwood, MA, USA). Figure 1 shows an optical image of the prepared samples after the tensile test.

##### Fourier Transforms Infrared Spectroscopy (FTIR)

The lignin-based composites were analysed in atmospheric air by an FTIR spectrometer (Nicolet™ iS™ 10, Thermo Fisher Scientific, Inc., Waltham, MA, USA), equipped with an attenuated total reflectance accessory without preparing KBr pellets, over a 400–4000 cm^−1^ range with a resolution of 2 cm^−1^ and 32 scans per sample. The background spectrum in the absence of samples was subtracted from the spectra of the individual samples.

##### Differential Scanning Calorimeter (DSC)

The sample was subjected to a differential scanning calorimeter (DSC Q 200, TA Instruments Inc. New Castle, DE, USA) with a heat–cool–heat mode to study the heat flow with a function of temperature. Nitrogen was used as purge gas during the experiment. Data were collected by heating the sample from 50 to 200 °C at a constant heating and cooling rate of 10 °C/min, and analysed using TA Instruments’ Universal Analysis Software (TRIOS Software v5.0.0, TA Instruments Inc. New Castle, DE, USA).

##### Thermogravimetric Analysis (TGA)

Thermal properties were analysed by thermogravimetric analyser TGA Q500, TA Instruments, New Castle, DE, USA. Ten milligrams of the samples was heated at a temperature range of 35 to 600 °C at a rate of 10 °C/min. The analysis required a nitrogen atmosphere with nitrogen flow rate of 20 mL/min. A graph was plotted on weight reduction against increasing temperature.

##### Scanning Electron Microscopy (SEM)

The surface morphology of torn samples was examined using a Hitachi S-3400N scanning electron microscope (Hitachi, Berkshire, UK) The acceleration voltage at the cathode was 15 kV. By using an Emitech K550X (Quorum Technologies Ltd., East Grinstead, UK) coater (600 s, 35 mA), the composite samples were sputter coated with gold at a pressure of 2 × 10^−1^ bar prior to scanning.

## 3. Results and Discussion

### 3.1. Effects of Esterification on Weight Percent Gain (WPG)

A WPG value of 30.21% was recorded after esterification. WPG is a convenient way to assess the extent of esterification in a sample. Esterification of lignin hydroxyl bonds involves a nucleophilic attack on the acyl carbon centre of the anhydride molecule by an ion pair of the lignin hydroxyl group [20].

### 3.2. Mechanical Properties of Lignin-Based Composites

**Figure 2:**
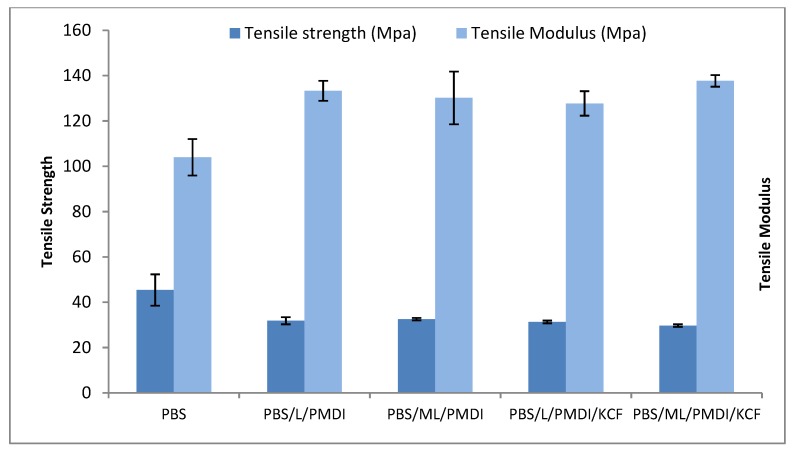
Mechanical properties of PBS polymer and lignin-based composites.

The tensile properties of the lignin-based composites are illustrated in Figure 2. It can be observed that the tensile strength of pure PBS (45.37 MPa) was higher compared to that of the lignin composites (29.65–32.55 MPa). In comparison to pure PBS, the addition of lignin and modified lignin reduced the tensile strength of the composites by 29.9% and 28.3%, respectively. Sahoo et al. [21] observed a similar trend when 30 wt % lignin was incorporated into PBS due to poor interfacial adhesion between the hydrophilic fillers and the hydrophobic polymer matrix. However, ML was found to have shifted to more hydrophobic basics, since maleic anhydrides remove most of the hydrophilic hydrogen bonding and hence better interfacial connection with hydrophobic PBS polymer matrix is observed with higher tensile strength (32.55 MPa). On the other hand, the incorporation of hydrophilic kenaf core fibers further reduces the tensile strength of the composites. Incompatibility of the fibers with the matrix and incomplete fiber wetting due to a lesser amount of PBS matrix in the composite are possible causes of tensile strength reduction. Nevertheless, in terms of tensile modulus, the values increased after incorporation of lignin and kenaf core fibers.

### 3.3. FTIR Analysis

**Figure 3:**
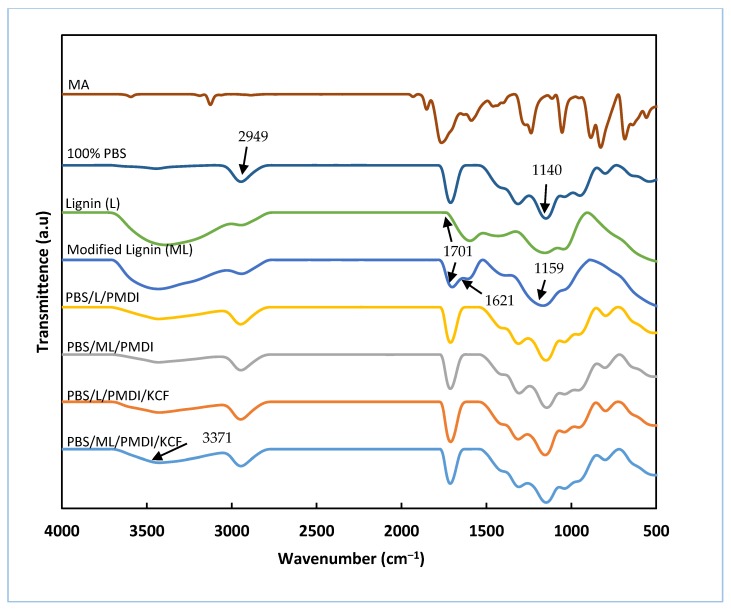
FTIR-ATR spectra of the KL and MA-LIG esterified lignin at a range of 4000 to 400 cm^−1^.

Figure 3 illustrates the infrared spectra of pure MA, pure PBS, pure lignin, MA-modified lignin, and composites made from MA-modified lignin and lignin. According to Zhao et al. [22], peaks found near 3429, 2949, 1715, and 1140 cm^−1^ corresponding to O–H stretching, C–H stretching, C=O stretching, and C–O–C stretching of the PBS, respectively. For unmodified lignin, the existence of a broad band near 3400 cm^−1^ corresponds to the O–H stretching vibration of hydroxyl groups [23]. Typical peaks corresponding to C–H stretching and C–H tensor were also recorded at bands around 2940 and 2840 cm^−1^, respectively. The intensity of the hydroxyl band reduced as the esterification reaction took place in the MA sample, indicating a decrease of lignin polarity as the hydroxyl groups were substituted by the carbonyl groups of MA. Anhydride modification of lignin was also confirmed by an intensified peak recorded around 1701 cm^−1^, which corresponds to the carbonyl stretching of ester and carboxyl groups in lignin. Compared to the strong peaks observed in maleated lignin, the peak is weak and almost undetectable in unmodified lignin [23]. Another confirmation of the occurrence of maleation reaction is a peak observed around 1621 cm^−1^ in modified lignin. It corresponds to the addition of a double bond C=C of maleic anhydride [24]. An intensified peak near 1159 cm^−1^ corresponding to C–O symmetric stretching also confirms the occurrence of maleation [25].

PBS/lignin and PBS/lignin/KCF composites basically have the same spectra as that of pure PBS, with the exception of the peak observed near 3371 cm^−1^ in the PBS/lignin/KCF composite. The peak near 3371cm^−1^ could be attributed to the stretching vibration of the aliphatic hydroxyl groups in the kenaf core fiber [26].

### 3.4. Thermal Analysis

#### 3.4.1. Differential Scanning Calorimetry (DSC)

All the samples undergo a heat-cool-heat cycle [27]. The second heating profile reflects the material’s thermal properties. DSC was used to study the thermal behaviour of lignin-based composites; details of glass transition (Tg), melting (Tm), and crystallization (Tc) temperature are listed in Table 2. The Tg of pure kraft lignin was found to be 148.3 °C and is slightly higher than that of modified lignin, since reactions with maleic anhydride destroy most of the intra- and inter-molecular hydrogen bonds, resulting in a softer structure at elevated temperature [28]. This finding is in line with previous studies, where the Tg of lignin was reported to be in the range of 110–150 °C [29,30]. However, the opposite was found in a study by Chen [20], who observed an enhancement of Tg for MA-modified lignin. The depression of Tg in this study may be due to the elimination of hydrogen bonds, leading to increased mobility within lignin molecules (found in the FTIR results), and quicker transition to a leathery state. However, the composite’s Tg was enhanced, when compared to the composite with pure kraft lignin. This is because modified lignin causes a higher restriction on polymer chain mobility in the composite; hence, a slightly higher Tg is recorded [23] Besides, the incorporation of kenaf core fibers have an insignificant influence on Tg. However, incorporation of kenaf fibers reduced the melting temperature of the composites. This is because the kenaf fibers create hindrance in the melting of the PBS composite [31].

#### 3.4.2. Thermo Gravimetric Analysis (TGA)

**Figure 4:**
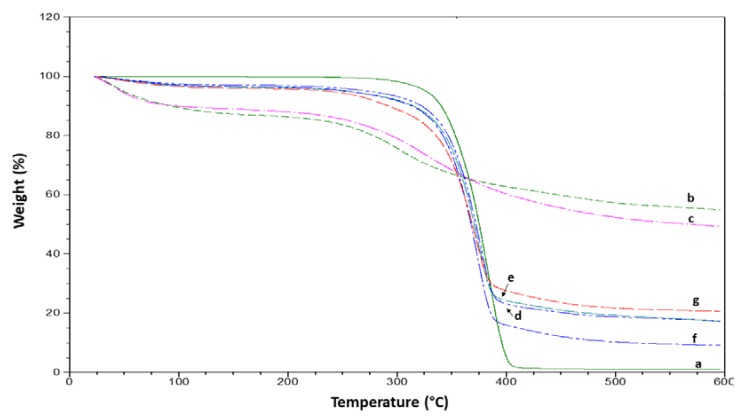
Thermo Gravimetric Analysis (TGA) curves for (**a**) neat PBS, (**b**) pure lignin, (**c**) modified lignin, (**d**) PBS/L/PMDI, (**e**) PBS/ML/PMDI, (**f**) PBS/L/PMDI/KCF, and (**g**) PBS/ML/PMDI/KCF.

The thermogravimetric (TG) curves obtained for pure kraft lignin and modified kraft lignin composites under nitrogen atmosphere are presented in Figure 4. Kraft lignin and its composites had a small weight loss (1–8%) below 100 °C due to gradual evaporation of moisture. The details of the thermal degradation temperature and final residual temperature at 600 °C were listed in Table 2. The weight loss of kraft lignin between 150 and 300 °C is attributed to the elimination of water, sulfur dioxide, carbon dioxide, formaldehyde, and formic acid, resulting from the degradation of the phenylpropane side chains at elevated temperatures [28].

As can be seen, the addition of kraft lignin enhanced the thermal stability of the PBS polymer composites. Between modified and unmodified kraft lignin, the former seems to impart better thermal stability to the PBS composite. At the same time, delayed onset thermal degradation temperature was also observed when the modified kraft lignin was incorporated. The modification of lignin with anhydrides substitutes the hydrophilic hydroxyl groups with covalently bonded maleic groups, rendering the surface more hydrophobic. Therefore, the enhancement in thermal properties might be attributed to the fact that modified lignin exhibits higher hydrophobic properties [20].

On the other hand, significant loss of mass and delayed onset temperature was observed during the TGA test for the composite samples. This could be mainly attributed to thermal degradation of the PBS polymer. However, when the lignin was incorporated, improvement in thermal stability was seen, as shown by the TGA curves. When comparing modified and pure kraft lignin composites, a lower onset temperature was recorded for the latter. Besides, the insertion of low thermal stability kenaf fibers in composites resulted in a lower onset temperature.

### 3.5. Surface Morphology

**Figure 5:**
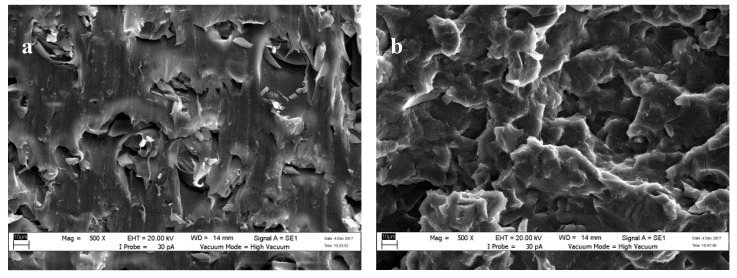
SEM micrographs of (**a**) PBS/L/PMDI, and (**b**) PBS/ML/PMDI.

The morphology of the PBS composite after incorporation of unmodified and modified lignin is shown in Figure 5. In the case of the PBS/lignin composite, agglomerated lignin was observed. Meanwhile, for the PBS/lignin-MA composite, lesser agglomerated lignin was observed, indicating better dispersion of maleated lignin in PBS. This explains the better tensile strength of the samples (described in the previous section). The hydroxyl groups of the lignin were reduced by the maleation and subsequently lead to a decrease in polarity as well as lower molecular interaction through hydrogen bonding. As a result, better dispersion of lignin in the PBS matrix could be attained [32].

## 4. Conclusions

In this study, modified lignin (ML) and kenaf core fibres (KCF) were incorporated into the PBS matrix to produce a lignin-based biocomposite. Lignin acted as a reinforcing agent, improving the thermal stability of the polymer composite. However, lower tensile strength was observed in the lignin-based biocomposite, compared to that of neat PBS. Compared with neat PBS, lignin incorporation increased the tensile modulus. Modification of lignin by maleic anhydride (MA) improved the dispersion of the lignin in the PBS matrix, as lesser agglomerated lignin was observed through SEM micrographs. Although incorporation of both types of lignin increased the thermal stability of the lignin-based composite, modified lignin showed a greater extent of improvement, compared to unmodified lignin. The results from this study suggest that MA-modified lignin has the potential to serve as a reinforcing agent for fabrication of thermally stable polymer composites.

## Figures and Tables

**Table 1:** Lignin-based composite fabrication and formulation.

Composites	Code	PBS (wt %)	Lignin (wt %)	PMDI (wt %)	KCF (wt %)
**PBS**	PBS	100	0	3	0
**PBS/Lignin/PMDI**	PBS/L/PMDI	70	30	3	0
**PBS/Modified Lignin/PMDI**	PBS/ML/PMDI	70	30	3	0
**PBS/Lignin/PMDI/KCF**	PBS/L/PMDI/KCF	60	30	3	10
**PBS/Modified Lignin/PMDI/KCF**	PBS/ML/PMDI/KCF	60	30	3	10

**Table 2:** Data obtained from differential scanning calorimetry (DSC) and thermo gravimetric analysis (TGA) results.

Samples	DSC			TGA		
	Tg, °C	Tm, °C	Tc, °C	Onset Temperature, °C	Final Degradation Temperature, °C	Residual (%)
PBS	-	115.3	-	350.3	410.1	1.1
MA	164.3	257.6	54.4	139.3	160.2	0.5
L	148.3	171.9	117.9	255.2	300.0	54.9
ML	146.9	175.1	175.1	264.9	310.1	49.3
PBS/L/PMDI	110.0	163.3	202.1	343.2	397.1	17.3
PBS/ML/PMDI	110.4	165.0	213.5	341.9	383.2	17.1
PBS/L/PMDI/KCF	110.4	163.7	215.8	341.6	390.2	9.07
PBS/ML/PMDI/KCF	110.8	154.1	203.1	335.3	395.0	20.6
